# Convergent and Discriminant Validities of SCBE-30 Questionnaire Using Correlated Trait–Correlated Method Minus One

**DOI:** 10.3389/fpsyg.2020.571792

**Published:** 2020-10-16

**Authors:** Marília Fernandes, António J. Santos, Marta Antunes, Carla Fernandes, Lígia Monteiro, Brian E. Vaughn, Manuela Veríssimo

**Affiliations:** ^1^William James Center for Research, ISPA – Instituto Universitário, Lisbon, Portugal; ^2^Instituto Universitário de Lisboa (ISCTE-IUL), CIS-IUL, Lisbon, Portugal; ^3^Family and Child Development, Auburn University, Auburn, AL, United States

**Keywords:** measurement invariance, correlated trait–correlated method minus one model, multiple informants, SCBE-30, social competence

## Abstract

Correlated trait–correlated method minus one was used to evaluate convergent and discriminant validity of Social Competence Behavior Evaluation questionnaire (Social Competence, Anger-Aggression, Anxiety-Withdrawal) between multiple raters. A total of 369 children (173 boys and 196 girls; *M*_*age*_ = 55.85, SD_*age*_ = 11.54) were rated by their mothers, fathers, and teachers. Results showed more convergence between parents than parent-teacher ratings. Mother-teacher share a common view of child behavior that is not shared with father. Parents had more difficulty distinguishing internalizing and externalizing behaviors (especially fathers). Measurement invariance across child sex was explored, results imply that differences between boys and girls were not due to measure. Girls (compare to boys) were described as more social competent by their fathers and teachers, while boys as more aggressive by mothers and teachers.

## Introduction

Social Competence Behavior Evaluation questionnaire (SCBE-30) is a rating scale on affective quality of children’s relationships with peers and significant adults, providing a standardized description of affect and behavior in context, discriminating behavioral-emotional problems and social adjustment (LaFreniere and Dumas, 1996). It has been used with children from 30 to 78 months, in different international settings, cross-sectional, and longitudinal research (LaFreniere et al., 2002). Correlated trait–correlated method minus one [CT-C(M−1)] (Eid, 2000; Eid et al., 2003), a multiple-trait by multiple-method (MTMM) approach, was used to examine convergent and discriminant validity of SCBE-30 between mother, father, and teacher.

Social Competence Behavior Evaluation questionnaire contains three scales with 10-items each: two distinct patterns of maladaptive behavior, Anger-Aggression (AA) and Anxiety-Withdrawal (AW); and one adaptive pattern, Social Competence (SC). Presenting good internal consistency across different countries, 0.87 for SC, 0.88 for AA, and 0.84 for AW (LaFreniere et al., 2002). It has been widely used in research, educational and clinical settings with demonstrated validity across cultural settings (Zupancic et al., 2000; Chen and Jiang, 2002; Kotler and McMahon, 2002; LaFreniere et al., 2002; Dumas et al., 2011; Klyce et al., 2011; Sette et al., 2014; Vasquez-Echeverria et al., 2016; Bárrig and Parco, 2017). However, most studies used only one rater (usually teacher) and few compared teacher with parents (mostly mother). Klyce et al. (2011) reported that although presenting identical factor structures, parent, and teachers showed low agreement when rating children’s behaviors. Munzer et al. (2018) also reported low parent-teacher concordance, especially among girls. Studies regarding children’s social behaviors ratings, also reported low agreement between informants (Achenbach et al., 1987; Winsler and Wallace, 2002; Konold et al., 2004; Reyes and Kazdin, 2005). Parents and teachers agree more on problem behaviors than on social skills, and more on externalizing than internalizing behaviors (Achenbach et al., 1987; Winsler and Wallace, 2002). Also, parents (more than teachers) rate children as having more behavior problems (Berg-Nielsen et al., 2012).

Rating scales implies that raters judge how a child typically behaves in comparison with others, constructing their valuations retrospectively (based on memory, which could be biased). It could also be influenced by their knowledge, beliefs and language, as well as by social values attributed to the behavior. It reflects raters’ ideas and representations (Uher et al., 2013). In rating scales, the item statements and answer categories involved encoding schemes (i.e., variables and values), they include adjectives from everyday language allowing raters to interpret the item meaning. However, they are often ambiguous and context-sensitive. Campbell and Fiske (1959) stated that psychological variable’s score reflects not only the psychological construct under consideration, but also systematic method-specific influences and they demonstrated the necessity to include at least two different methods (that should converge when measuring the same trait) to separate trait from method influences.

Most studies assessed inter-rater agreement by correlating ratings, but even highly correlated data could present poor agreement. MTMM analysis allows the study of multiple traits measured by multiple methods and evaluate convergent and discriminant validity more robustly (Lance et al., 2002). Confirmatory factor analysis (CFA) is one of the most common methods to analyze MTMM data (Eid et al., 2006) and allows to calculate correlations among latent factors rather than observed variables, accounting for measurement error. According to Eid et al. (2008) when selecting a CFA model for MTMM analysis the key issue is the type of methods in the model. Methods can be either interchangeable (i.e., all raters have same access to the target, therefore the target is rated from the same perspective) or structurally different (all raters have different access to the target, responding from different perspectives). We selected CT-C(M−1) to compare and contrast our structurally different methods, with each SCBE-30 trait being represented by multiple indicators. Parents and teachers were considered structurally different and fixed for each child. We specifically selected different raters (mother, father, and teacher) to evaluate same child on multiple traits (SC, AA, AW) recognizing that each one has a unique perspective and access to partially overlapping information of child behavior. Since CT-C(M−1) model is not symmetrical the meaning of the parameters of the model depends on the method chosen as the reference standard (Geiser et al., 2008). One method is selected as reference (reference rater) and its true-scores indicators are used to predict true-scores indicators of non-reference (other raters). If we choose mother ratings as the reference method in the CT-C(M−1) model, we are evaluating the convergence of mother ratings with teacher ratings and father-ratings. This analysis will not show how teacher ratings and father-ratings converge with each other. A second analysis is needed in which teacher ratings is the reference method and father-ratings are one of the non-reference method, or father-ratings is the reference method and teacher ratings is one of the non-reference method. Convergence between methods is inferred by consistency coefficients of non-reference methods, reflecting shared variance. Whereas method-specific coefficient reflects the proportion of variance in non-reference methods that is not predicted by true-score of reference method. For a multidimensional rating scale as SCBE-30, subscale convergent validity is inferred when there are relatively high monotrait–heteromethod correlations (same subscale across different raters) whereas discriminant validity is inferred by relatively low heterotrait–monomethod correlations (different subscales within raters), and method effects (raters effects) are inferred when correlations for subscales within a method are larger than correlations across methods but within traits (Lance et al., 2002). Since reference method selection influences trait and method factors meanings (Geiser et al., 2008) three different analyses were conducted. In first analysis (analysis1) teacher was used as reference, in the second (analysis2) mother, and father in the third (analysis3). Conducting these complementary analyses allowed comparations between all raters (analysis1, 2, or 3), but also to contrast teacher with parents’ ratings (analysis1), mother with teacher/father (analysis2), and father with teacher/mother (analysis3).

Using a multiple-group confirmatory factor analysis (MG-CFA) sex measurement invariance (MI) was tested. Literature points some differences although to our knowledge only Munzer et al. (2018) evaluated MI. In most studies girls are rated higher than boys on SC and lower on AA (LaFreniere et al., 2002; Masataka, 2002; Venet et al., 2002; Torres et al., 2014; Vasquez-Echeverria et al., 2016). No sex differences we reported regarding AW, except for two studies (Chen and Jiang, 2002; Blair et al., 2004) where boys were rated higher by their teachers. Bárrig and Parco (2017) found no sex differences.

Based on exiting data, we expect more convergence between mother–father ratings than parent–teacher ratings and for externalizing more than internalizing problem behavior.

## Materials and Methods

### Participants

Participants were parents and teachers of 369 children (173 boys and 196 girls, ages ranged from 32 to 78 months, *M* = 55.85, SD = 11.54, 55.8% firstborns, and 63.7% had siblings). All attended public preschools. Each one of the 45 classes had on average 20 children (19 to 24), all families were invited to participate (one child per household).

Most parents were married or cohabiting (95.1%). Mothers age ranged between 21 and 47 years (*M* = 33.53; SD = 6.70), and fathers from 23 to 55 (*M* = 35.97; SD = 7.28). Mothers education level varied between 4 and 21 years (*M* = 11.94; SD = 4.59) and fathers between 1 and 19 (*M* = 10.34; SD = 4.64). Most parents work full-time (mother *M* = 38.39 h; SD = 7.34, 20.6% unemployed; fathers *M* = 41.60; SD = 7.23, 9.8% unemployed).

All 45 participating teachers were female, with age between 41 and 50 years (*M* = 44.82; SD = 2.75). All had a university degree in early education and 21 to 25 years of experience.

## Procedure

Stratified random sampling was used to select, the population was divided into 20 groups corresponding to Portugal’ regions. Within each region, a random number table was used to determine the schools to be contacted. From total of 63 schools, 30 consented to participate, and 45 classes contribute to the study. Parents were asked to complete questionnaires independently. Forty-one percent of the questionnaires were returned with all the information, and the consent for teachers to report on the child behavior (one per family). Teachers rated consented children (middle to the end of the year to guarantee that they were well acquainted with the child), resulting in 369 ratings with complete (usable) sets of mother, father, and teacher ratings. Only completed sets of mother, father, and teacher were analyzed.

### Instrument

#### Social Competence and Behavior Evaluation Scale

Evaluates patterns of social competence, emotion regulation and expression, as well as adjustment difficulties in children between 30 to 78 months (LaFreniere and Dumas, 1996). It intends to describe behavioral tendencies of socialization rather than to classify children. It has three 10-items scales that allow the assess to the overall quality of the child’s adaptation including their strengths as well as their weaknesses: (1) SC, referring to prosocial behaviors; (2) AA, referring to externalizing behaviors; and (3) AW, referring to internalizing behavior. Responses range from 1 (never) to 6 (always). SCBE-30 was translated from the original English version into Portuguese following the procedures outlined by “Committee Approach” (Brislin, 1980).

### Data Analysis

For missing Little’s MCAR statistic was computed (χ^2^ = 1402.56, df = 1344, *p* = 0.13) and estimation maximization algorithm (EM) was used. Confirmatory factor analyses (CFA) and MI were performed using the *R* packages *Lavaan* (Rosseel, 2012), *SemTools* (Jorgensen et al., 2018) to evaluate SCBE-30 three factor model fit. Given data ordinal nature, we used Robust Weighted Least Squares (RWLS) (Flora and Curran, 2004) and configural invariance was evaluated using three robust indices (Hu and Bentler, 1999; Brosseau-Liard et al., 2012): robust Comparative Fit Index (CFI, ≥0.95 good and ≥0.90 acceptable); robust Root Means Square Error Approximation (RMSEA, ≤0.06 good and ≤0.08 acceptable) and Weighted Root Mean Square Residual (WRMR, ≤1.0 good, with lower values indicating better fit; Yu and Muthén, 2002). For model fit improvement, factor loadings were considered (<0.40 poor) (Hair et al., 1998). We considered a global model (M1, not distinguishing who answered the questioners – mothers, fathers, or teachers). Since we were interested on comparing and contrasting mother’s, father’s, and teacher’s responses we modify the model to include that distinction (M2). Because the same child was being reported by parents and teacher, we explore the dependency of the observations by correlating the residual covariance between same indicator across parents and teachers (M3).

Correlated trait–correlated method minus one model (Eid et al., 2008) was selected to test for MI across our structurally different raters, comparing and contrasting them against each other (Eid et al., 2006). This model implies that the trait cannot be measured independently of the method (rater), with each observed variable (item) representing a trait-method unit. By contrasting different methods against each other the convergent validity of the different methods can be determined. CT-C(M−1) includes two types of latent variables: a reference factor, representing the trait as measured by the reference method; and a method factor, representing the residual variance in the non-reference method (not shared with the reference factor within the same trait). Non-reference methods are contrasted against the reference factor. Since method factors are defined as regression residuals, reference and method factors for the same trait are uncorrelated. To create our models, all indicators of reference method (teacher in analysis1, mother in analysis2, and father in analysis3) were linked to appropriate trait factors but not to any method factor. For non-reference methods (mother and father ratings in analysis1; father and teacher ratings in analysis2, mother and teacher ratings in analysis3) indicators were linked to appropriate trait factors and method factors. The trait factors were correlated with each other and same happened to method factors, whereas method and trait factors were assumed to be uncorrelated. High trait loadings of non-reference method and comparatively low method loadings of non-reference methods indicate more agreement with reference method. Method factor will be the common residual factor, representing the proportion of a trait measured by non-reference method that cannot be predicted by reference true-scores. Proportion of variance shared with reference model is given by square standardized loadings of non-reference indicators onto reference factor. The rater-specific variance that cannot be predicted by true-score variable of the indicator measured by reference method is given by squared standardized loadings of non-reference indicators on method factors (method-specific coefficient). Total reliable variance of an indicator (reliability coefficient) is given by the sum of the consistency plus method-specific coefficients (Eid et al., 2003).

Sex invariance was tested using MG-CFA, we analyzed configural invariance (factor structure with same items being associated with same construct), metric invariance (raters use questionnaires scales in similar, presenting equivalent loadings), and scalar invariance (equivalent items thresholds) (Geiser et al., 2014). When differences in fit indices (ΔCFI and ΔRMSEA) between a model and the (preceding) less constrained model was ≤0.01 for ΔCFI and ≤0.015 for ΔRMSEA level of MI was achieved (Chen, 2007). Latent mean differences between child sex (for all raters on SCBE-30 dimensions) was compared using a full scalar invariance model as the baseline.

## Results

### Confirmatory Factor Analyses of SCBE-30

Prior to our main analyses we examined items distributions (see Table 1). To evaluate model fit and consistency with data we performed a CFA, using RWLS. As showed in Table 2, initial model (M1) using all 30 items organized in three factors (not considering different raters) did not present an acceptable fit. For model improvement, we had in consideration that there were three different raters (M2), and in M3 we added residual covariances between raters’ related items as they were describing the same child (see Table 1 for residual covariance). In M4 we dropped item8 “sad” was left skewed for all raters (mother *Sk* = 3.20 *Ku* = 13.09; father *Sk* = 2.28 *Ku* = 6.21; teacher *Sk* = 2.65 *Ku* = 9.58). In following models, we eliminated two items presenting low factor loadings (*λ* < 0.40) for all raters: M5 we dropped item6 “worries,” values were unexpectedly high (specially for parents) and modification indices suggest better fit on SC; M6 we removed item13 “negotiates solutions to conflicts,” values were low and modification indices suggest better fit on AA or AW. In the following models we gradually eliminated two more items presenting low factor loadings for two of the raters: M7 we deleted item1 “neutral expression” that presented low factor loading for fathers and teachers; M8 we dropped item2 “tired” that presented low factor loadings for parents, an acceptable fit (robust CFI = 0.91, RMSEA = 0.038, and WRMR = 1.35) was achieved.

**TABLE 1:** SCBE-30 items distributions considering mothers, fathers, and teachers (*N* = 369).

		Global			Mother			Father			Teacher			σ		
	Item	M	SD	λ	M	SD	λ	M	SD	λ	M	SD	λ	MF	MT	FT
SC	13	2.71	1.29	0.38	2.77	1.28	0.43	2.83	1.31	0.44	2.52	1.25	0.38	0.42***	0.15**	0.15**
	15	3.14	1.21	0.57	3.18	1.14	0.51	3.30	1.13	0.52	2.93	1.32	0.64	0.30***	0.06	0.11*
	17	3.97	1.33	0.66	4.14	1.31	0.66	4.15	1.30	0.56	3.63	1.32	0.75	0.23***	0.01	0.01
	19	3.68	1.38	0.68	3.91	1.37	0.70	3.92	1.29	0.69	3.21	1.35	0.74	0.36***	0.06	0.04
	20	3.58	1.47	0.63	3.68	1.50	0.60	3.44	1.45	0.57	3.62	1.46	0.68	0.36***	−0.07	−0.06
	22	4.37	1.51	0.63	4.53	1.55	0.60	4.54	1.48	0.63	4.03	1.46	0.72	0.30***	0.11*	−0.01
	24	4.27	1.38	0.68	4.24	1.40	0.66	4.35	1.31	0.60	4.22	1.43	0.78	0.34***	−0.01	0.02
	26	3.90	1.41	0.57	3.93	1.41	0.51	3.79	1.37	0.55	3.98	1.46	0.61	0.40***	(-0.07	0.00
	27	4.11	1.36	0.71	4.09	1.32	0.63	3.98	1.25	0.68	4.26	1.47	0.79	0.26***	−0.08	0.01
	30	5.15	1.13	0.64	5.24	1.18	0.55	5.21	1.09	0.61	4.99	1.11	0.69	0.28***	0.10	−0.08
AA	3	2.28	1.14	0.66	2.34	1.09	0.61	2.33	1.07	0.62	2.16	1.25	0.71	0.30***	−0.10	−0.09
	4	2.62	1.28	0.70	2.93	1.19	0.60	2.92	1.22	0.59	2.01	1.19	0.78	0.35***	−0.05	−0.05
	5	2.42	1.24	0.80	2.51	1.18	0.69	2.61	1.20	0.72	2.14	1.28	0.85	0.20***	−0.06	−0.09*
	10	2.17	1.23	0.70	2.22	1.21	0.64	2.43	1.26	0.67	1.85	1.14	0.78	0.20***	−0.06	−0.01
	11	1.67	0.97	0.62	1.63	0.95	0.56	1.63	0.87	0.58	1.74	1.08	0.76	0.34***	−0.08	0.06
	16	1.66	0.96	0.66	1.56	0.90	0.68	1.54	0.77	0.61	1.87	1.15	0.85	0.41***	0.27***	0.29***
	18	2.11	1.07	0.65	2.00	0.92	0.60	2.06	0.99	0.60	2.28	1.26	0.85	0.31***	0.11*	0.13*
	25	1.77	1.13	0.71	1.86	1.10	0.61	1.95	1.19	0.69	1.52	1.06	0.86	0.35***	0.04	0.11*
	28	2.42	1.16	0.66	2.79	1.06	0.49	2.78	1.05	0.50	1.69	1.00	0.82	0.20***	0.03	−0.01
	29	2.32	1.31	0.68	2.60	1.25	0.51	2.58	1.26	0.57	1.78	1.24	0.84	0.35***	0.08	0.05
AW	1	2.22	1.45	0.38	2.17	1.49	0.58	2.22	1.51	0.48	2.29	1.36	0.31	0.52***	0.09	0.08
	2	1.83	0.87	0.49	1.82	0.82	0.40	1.86	0.84	0.43	1.81	.94	0.54	0.35***	−0.09	−0.06
	6	3.02	1.37	0.11	3.19	1.36	0.28	3.27	1.30	0.13	2.60	1.35	−0.35	0.54***	0.00	0.02
	7	2.36	1.27	0.70	2.40	1.20	0.66	2.57	1.27	0.69	2.12	1.30	0.77	0.37***	−0.04	0.01
	8	1.35	0.70	0.72	1.29	0.67	0.65	1.33	0.63	0.63	1.43	.77	0.82	0.32***	−0.04	−0.08
	9	1.79	1.03	0.77	1.88	1.04	0.70	1.86	1.01	0.65	1.62	1.02	0.86	0.30***	−0.07	−0.02
	12	1.56	0.94	0.68	1.63	1.03	0.63	1.69	1.00	0.60	1.35	.75	0.79	0.37***	−0.02	−0.02
	14	1.39	0.75	0.79	1.41	0.75	0.76	1.40	0.70	0.72	1.37	.78	0.84	0.21***	0.10	0.19***
	21	1.45	0.95	0.64	1.41	0.89	0.67	1.55	0.97	0.61	1.39	.98	0.63	0.29***	0.19*	0.08
	23	1.88	1.19	0.53	2.00	1.21	0.51	2.10	1.28	0.49	1.54	.99	0.59	0.42***	0.07	0.04

λ, factor loadings, for global loadings we used Model 1 and for each rater loadings we used Model 2; (, σ (= residuals covariances using Model 3 (MF-mother/father, MT-mother/teacher, FT-father/teacher); *p < 0.05; **p < 0.01; ***p < 0.001.

**TABLE 2:** Robust fit indices for SCBE-30 CFA models.

Model	Items deleted	RWLS	df	*p*	CFI	TLI	RMSEA (90 % CI)	SRMR	WRMR
M1		4298.01	420	<0.001	0.78	0.76	0.094 (0.091; 0.096)	0.10	3.04
M2		6771.34	3879	<0.001	0.81	0.81	0.045 (0.043; 0.047)	0.09	1.70
M3		6002.41	3789	<0.001	0.86	0.85	0.040 (0.038; 0.042)	0.09	1.53
M4	8	5651.72	3531	<0.001	0.86	0.85	0.040 (0.038; 0.042)	0.09	1.53
M5	8 and 6	5032.62	3282	<0.001	0.88	0.88	0.038 (0.036; 0.040)	0.08	1.44
M6	8, 6, and 13	4519.18	3042	<0.001	0.90	0.89	0.036 (0.034; 0.039)	0.08	1.37
M7	8, 6, 13, and 1	4224.21	2811	<0.001	0.90	0.90	0.037 (0.035; 0.039)	0.08	1.36
M8	8, 6,13,1, and 2	3937.01	2589	<0.001	0.91	0.90	0.038 (0.035; 0.040)	0.08	1.35

**N* = 369. M1, all 30 items, three dimensions model; M2, all 30 items, three dimensions and considering the different raters; M3, all 30 items, three dimensions with residual covariances between mothers, fathers and teachers related items. RWLS, Robust Weighted Least Squares; CFI, Robust Comparative Fit Index; TLI, Robust Tucker-Lewis-Index; RMSEA, Robust Root Mean Square of Approximation; SRMR, Standardized Root Mean Square Residual; WRMR, Weighted Root Mean Square Residual.*

### Measurement Invariance Across Mother, Father, and Teacher

In first CT-C(M−1) we used teachers as reference method as major differences were excepted between parents-teacher (see Geiser et al., 2012 for guidelines). We used M8 but it did not converge, three items could not be obtained and were excluded (item3 “easily frustrated,” item4 “angry when interrupted” and item5 “irritable” all from AA) [CFI = 0.91, TLI = 0.89; RMSEA = 0.042 (0.040; 0.045), SRMS = 0.063, WRMR = 1.18]. Results showed low trait loadings (<0.40, except for item16 “hits”) and comparatively high (>0.60) method factor loadings suggesting low agreement between parents and teacher. These interpretations were confirmed by the low reliabilities (SC: mother 0.28 to 0.52, father 0.29 to 0.48; AA: mother 0.28 to 0.59, father 0.29 to 0.55; AW: mother 0.35 to 0.64, father 0.29 to 0.57) and because method-specific coefficients were higher than consistency coefficients for all items. We found high associations (0.71 to 0.82) between parents when considering the same trait, showing that parents share a common view of child behavior that is not shared with teachers. Since method effect goes in the same direction for both parents (positive correlations), when mothers over or underestimated child behavior (comparing to teacher) fathers do the same. The absolute values of correlations between method factors belonging to same method but different traits are mostly low (all < 0.20) for parents except when relating AW with AA (0.36 for fathers and 0.50 for mothers) these traits could be method biases, when parents overestimate AW also overestimate AA.

In analysis2, with mother as reference method, robust fit indices were good [CFI = 0.95, RMSEA = 0.033 (0.030; 0.036), SRMS = 0.057, WRMR = 0.95]. As in previous analysis, trait loadings within teacher trait factor were weak (<0.40) (except item16 “hits”) comparative to method factor loadings (all > 0.60). However, father’s trait loadings were above 0.52 (except for item28 “opposes”) and method factor presented lowest values. Indicators had larger consistency than method specificity coefficients, meaning that (as in analysis1) there is good support for mother-father, but not for mother-teacher. There was no significant association between method factors belonging to same trait showing that father and teacher do not share a common view of child behavior besides the one shared in mother. Absolute values of associations between method factors belonging to same method but different traits were significant for teachers (0.18 to 0.51). AW and AA were positively correlated (but low) besides correlation between SC and these two affective traits were negative. Comparing to mothers, teachers who overestimate AW also overestimate AA, and when overestimated AA or AW they underestimate SC. For fathers only the relation between AW and AA was significant (*r* = 0.58) meaning that (comparing to mothers) fathers who under or overestimate AW also do it for AA.

Finally, analysis3 with father as reference, robust fit indices were good [CFI = 0.94, RMSEA = 0.034 (0.031; 0.037), SRMS = 0.057, WRMR = 0.96]. As in previous analysis, teacher’s trait loadings within factor were weak (all < 0.40) although strong (all > 0.60) when considering method factor loadings. Again, when we compare parents most of trait loadings were good, indicators had a larger consistency coefficient than method specificity coefficient (except item30 “pleasure in own accomplishments,” items 9“inhibited,” 14 “isolated,” and 16 “hits”), there is good support for convergent between parents, but not for fathers and teachers. Mother and teacher share a common view of the child that is not shared with father, specifically a positive and significant association (although low) for SC (*r* = 0.18) and for AA (*r* = 0.26), meaning that when mothers over or underestimated child behavior (comparing to fathers) teachers do the same. The absolute values of the associations between method factors belonging to same method but different traits are significant for teachers (0.13 to 0.53), for mothers only relation between AW and AA were significant (0.69), these traits could be method biases, teachers and mothers who overestimate a child’s AW also overestimate AA. For teachers, correlations between SC and those two affective traits were negative, meaning that teachers who overestimate a child’s AW or AA also underestimate SC.

### Measurement Invariance Between Boys and Girls

To test for MI across child’s sex, we performed a MG-CFA for each rater separately. There were same cross table zeros therefore we collapse few items’ categories (Higgins, 2004). For teacher, we collapse category 6 into 5 for item12 “inactive” (girls 0; boys 1), item23 “unnoticed” (girls 2; boys 0), item28 “defiant” (girls 1; boys 0), category 5 into 4 for item14 “isolated” (girls 0; boys 3), and category 2 into 3 for item30 “pleasure in accomplishments” (girls 0; boys 7). Since MG-CFA results were below cut point metric (ΔCFI = 0.001; ΔRMSEA = −0.001) and scalar (ΔCFI = −0.001; ΔRMSEA = 0.008) invariance was achieved. For mother, we collapse category 6 into 5 for item6 “hits” (girls 1; boys 0), item18 “conflict” (girls 1; boys 0), item4 “isolated” (girls 0; boys 3), and category 2 into 3 for item30 “pleasure in accomplishments” (girls 3; boys 0). Results were below cut point metric (ΔCFI = 0.001; ΔRMSEA = −0.003) and scalar (ΔCFI = −0.004; ΔRMSEA = −0.006) invariance was achieved. For father, we collapse category 6 into 5 for item9 “inhibited” (girls 0; boys 1), item18 “conflict” (girls 0; boys 2), category 5 into 4 for item14 “isolated” (girls 0; boys 1). Results were below the cut point metric (ΔCFI = −0.005; ΔRMSEA = 0.003) and scalar (ΔCFI = 0.001; ΔRMSEA = 0.005) invariance was achieved. Suggesting differences in the underlying latent trait rather than in the measure for all raters. Based on the establishment of the full scalar invariance across child sex, latent mean difference between SCBE-30 for the different raters was explore. Results showed that girls had significant higher SC than boys for all raters with a small effect for parents and a medium effect for teachers (Mother, *M*_*girls*_ = 4.18, SD_*girls*_ = 0.69; *M*_*boys*_ = 4.03, SD_*girls*_ = 0.69, *t*(367) = −2.12, *p* < 0.05; Cohen’s *d* = −0.22) (Father, *M*_*girls*_ = 4.15, SD_*girls*_ = 0.67; *M*_*boys*_ = 4.00, SD_*girls*_ = 0.64, *t*(367) = −2.23, *p* < 0.05; Cohen’s *d* = −0.23) (Teacher, *M*_*girls*_ = 4.07, SD_*girls*_ = 0.82; *M*_*boys*_ = 3.65, SD_*girls*_ = 0.87, *t*(367) = −4.76, *p* < 0.001; Cohen’s *d* = −0.50). No differences were found regarding AW. For AA, results showed that boys had significant higher scores than girls for mothers and teachers, no differences were found considering fathers ratings (Mother, *M*_*girls*_ = 1.99, SD_*girls*_ = 0.49; *M*_*boys*_ = 2.15, SD_*girls*_ = 0.50, *t*(367) = 3.13, *p* < 0.01; Cohen’s *d* = 0.33) (Teacher, *M*_*girls*_ = 1.66, SD_*girls*_ = 0.76; *M*_*boys*_ = 1.97, SD_*girls*_ = 0.89, *t*(367) = 3.58, *p* < 0.001; Cohen’s *d* = 0.39).

### Social Competence, Anger-Aggression, Anxiety-Withdrawal: Associations Within and Between Raters

For parents, we found a positive relation between AA and AW (mothers *r* = 0.33, *ρ* < 0.001; and fathers *r* = 0.30, *ρ* < 0.001) for teachers this relation was negative (*r* = −0.14, *ρ* < 0.01). Associations between AA and SC were negative for all raters (mothers *r* = −0.11, *ρ* < 0.05; fathers *r* = −0.17, *ρ* < 0.001: and teachers *r* = −0.46, *ρ* < 0.001). AW and SC were negative associated but only for teachers (*r* = −0.24, *ρ* < 0.001). Correlations between raters were positive between all raters for SC (0.20 to 0.61) and AW (0.12 to 0.49) specially between parents. For AA, we found positive associations between parents (*r* = 0.47, *ρ* < 0.001) and between mother and teachers (*r* = 0.19, *ρ* < 0.001). Both parents described children as more SC but also as more maladapted then teachers do, and fathers perceived child behavior as more AW than mothers do (see Table 3).

**TABLE 3:** Mean differences between raters considering SC, AW, and AA dimensions.

	Mother		Father		Teacher				
	*M*	SD	*M*	SD	*M*	SD	MF	MT	FT
SC	4.10	0.85	4.08	0.81	3.87	0.98	*ns*	*t*(368) = 3.96***; *d* = 0.25	*t*(368) = 3.45***; *d* = 0.23
AW	1.79	0.68	1.89	0.64	1.64	0.72	*t*(368) = −2.93**, d = 0.15	*t*(368) = 3.07**, *d* = 0.21	*t*(368) = 5.32***, *d* = 0.37
AA	2.10	0.68	2.03	0.63	1.70	0.80	*ns*	*t*(368) = 8.17***, *d* = 0.54	*t*(368) = 6.70***, *d* = 0.45

*ns*, no significant; *p < 0.05; **p < 0.01; ***p < 0.001; *d*, Cohen’s *d*; SC, Social Competence; AW, Anxiety-Withdrawal; AA, (=Anger-AggressionA; MF, mother/father differences; MT, mother/teacher differences; FT, father/teacher differences.

## Discussion

Our study presents important methodological contributions: child behavior was described by three raters, not only by their teacher but also by their parents (including father’s perspective); and we used CT-C(M−1) to compare and contrast raters.

Social Competence Behavior Evaluation questionnaire three-factor structure was analyzed taking all raters simultaneously, considering dependency of observations and ordinal nature of data. Factor structure remained the same, though some items were excluded (items 8, 6, 13, 1, and 2). Item8 “sad” was excluded due to normality problems, all raters described children as usually not sad or depressed, which is expectable in a non-clinical sample as ours. Sette et al. (2014) also excluded this item based on cross-loadings on both AW and AA. Item6 “worries” was excluded due to low loadings for all raters, values were unexpectedly high (specially for parents) and modification indices suggest a better fit on SC. This might be due to a translation issues, raters could be linking Portuguese word “*preocupa-se*” more in a sense of “being thoughtful,” which is more related to SC. Same word was used by Vasquez-Echeverria et al. (2016) and item was excluded due to low factor loading. Brazilian study (Brigas and Dessen, 2002) used “*desasossegado*,” item was also excluded as in other non-English studies (e.g., Butovskaya and Demianovitsch, 2002; Sette et al., 2014). Item13 “negotiates solutions to conflicts,” presented low factor loadings for all raters and modification indices suggest better fit on AA or AW. Raters could be reporting how frequently child is involved in conflicts rather than the ability to negotiate solutions with others (even if not frequently involved in conflicts). Vasquez-Echeverria et al. (2016) also excluded considering it distinct from the rest of SC items.

A strong agreement between both parents was found, and low agreement when comparing parents with teacher (for all SCBE-30 dimensions). Previous study by Klyce et al. (2011) that analyzed teacher’s and parent’s (92.8% mothers) ratings on SCBE-30, suggest that low agreement found between raters could be related with context, teachers might be concerned with disruptive behavior in classroom, whereas parents might have more opportunities to notice children coping positively when facing affective/emotional challenges. Our results showed that parents share a common view of child behavior that is not shared with teachers. Different opportunities, concerns, knowledge, expectations, and experiences could influence their perceptions. Parents observe qualitatively different behaviors and have greater familiarity with their children’s verbal and nonverbal cues in multiple contexts. Whereas, teachers only have one context, although multiple children to compare with, and more academic knowledge related to child development. A meta-analysis regarding behavioral/emotional problems (Achenbach et al., 1987) reported significant higher correlations for similar informants (e.g., mother-fathers) whereas ratings from different types of informants (e.g., parents, teachers) were less correlated. A more recent meta-analysis (Renk and Phares, 2004) regarding social competence reported modest average weighted effect size for both mothers-fathers and parents-teacher’s ratings.

Another interesting finding is the higher agreement between mother-teachers (comparing to father-teachers). Mother and teacher share a common view of child behavior that is not shared with father, whereas father-teacher do not share a common view besides the one that is shared with mother. This could be due to how schools include fathers, acting in a gender-type manner (Klinman, 1986) with teachers talk to about children manly with mothers. Also, typically fathers invest less time and effort (Torres et al., 2014) and might not have same opportunities to observe behaviors. It could also be related to individual differences in tolerance for various behaviors (Youngstrom et al., 2000), they might differ in perceiving occurrence or severity of behaviors. Or it could be related with gender bias since all teacher were females.

A recent meta-analysis (Rescorla et al., 2014) reported that parent–teacher agreement was higher for externalizing and attention problems than for internalizing. Our results suggest that parents have more difficulty on distinguishing internalizing and externalizing behaviors (especially fathers), associating higher AA exhibition with higher AW behaviors. Whereas teacher seems distinguish those behaviors and described children with higher SC scores as the ones who also presented less AW and AA behaviors. The ways in which raters regard social or problem behavior may contribute to rating differences. Previous study using parent, teacher rating, and observational data, found that correspondence between ratings and independent observations regarding problem behavior varied as a function of type of problem (internalizing/externalizing). Specifically, only parents’ ratings on internalization predicted observed isolation and withdrawal. Whereas, only teachers’ ratings on externalization predicted observed disobedient and aggressive actions (Hinshaw et al., 1992).

Our results suggest that differences between boys and girls are not due to measurement variance. Girls (more than boys) were described as more social competent, while boys (more than girls) were described as more aggressive. These results are consistent with literature (LaFreniere et al., 2002; Diener and Kim, 2004; Torres et al., 2014; Vasquez-Echeverria et al., 2016). Parents and teachers may be more aware of boys’ misbehavior, and more tolerant with girls, they could expect boys having more problem behaviors (Berg-Nielsen et al., 2012) and girls to display more socially competent behaviors (Birch and Ladd, 1998; Coolahan et al., 2000).

We recognize some study limitations. Analysis was based in parents and teacher perceptions of children’s social behavior rather than in direct observation, which may yield different interpretations. Additional bias factor could be present and were not controlled (e.g., fatigue, response bias, or contrast effects since parents rated only one child while teachers rated more children). Our sample presented 6% of multivariate outliers (*Mahalanobis distance* by χ^2^ (29) = 58.30, *p* < 0.001). Also, an exploratory structural equation modeling (ESEM) could represent better option since CFA might fail to meet standards of good measurement (e.g., goodness-of-fit, MI, and well-differentiated factors) (Marsh et al., 2020). It should be noted that for our sample (although for a short number of items) a collapsing categories technique has used to test for MI across child’s sex, replication is needed to examine the robustness our findings.

Future research could benefit from multilevel analysis with teachers’ ratings nested within classrooms and also explore in more detail discrepancy between raters (e.g., items analysis) to identify specific behaviors or contexts. It is important to consider how discrepancy between parents and teacher impact their communication and how it could affect children.

## Data Availability Statement

The raw data supporting the conclusions of this article will be made available by the authors, without undue reservation.

## Ethics Statement

The studies involving human participants were reviewed and approved by the ISPA Ethics Committee. The patients/participants provided their written informed consent to participate in this study.

## Author Contributions

MV, AS, and MF: conception of the work. MA, CF, MF, and LM: data collection. MF: data analysis and drafting the manuscript. MF, MV, AS, and BV: data interpretation and edit the manuscript. All authors read and commented on the manuscript.

## Conflict of Interest

The authors declare that the research was conducted in the absence of any commercial or financial relationships that could be construed as a potential conflict of interest.
