# Supplementary material for: Convergent and Discriminant Validities of SCBE-30 Questionnaire Using Correlated Trait–Correlated Method Minus One
Source: Front Psychol. 2020 Oct 16;11:571792. doi: 10.3389/fpsyg.2020.571792 (PMC7596166; doi:10.3389/fpsyg.2020.571792)
Supplement: Supplementary file 1 [file Table_1.pdf]

Suplementary table 1. CT-C(M-1) Results: Standardized Loading Parameters, Reliability, Consistency and Method Specific

Teacher as Reference method

		T	M	1	2	3			T	M	1	2	3			T	M	1	2	3
Social Competence	**Mother**						Anxiety-Withdraw	**Mother**						Anger-Aggressive	**Mother**					
	Item13	-	-	-	-	-		Item01	-	-	-	-	-		Item03	-	-	-	-	-
	Item15	.25	.46	.28	.06	.21		Item02	-	-	-	-	-		Item04	-	-	-	-	-
	Item17	.24	.61	.43	.06	.37		Item06	-	-	-	-	-		Item05	-	-	-	-	-
	Item19	.33	.64	.52	.11	.41		Item07	.05	.68	.47	.00	.47		Item10	.11	.62	.39	.01	.38
	Item20	.12	.64	.42	.01	.41		Item08	-	-	-	-	-		Item11	.03	.65	.43	.00	.43
	Item22	.34	.53	.39	.11	.28		Item09	.08	.73	.54	.01	.53		Item16	.46	.61	.59	.21	.37
	Item24	.27	.62	.45	.07	.38		Item12	.10	.63	.41	.01	.40		Item18	.32	.57	.43	.10	.32
	Item26	.01	.58	.34	.00	.34		Item14	.28	.75	.64	.08	.56		Item25	.24	.62	.44	.06	.38
	Item27	.12	.65	.43	.01	.42		Item21	.21	.64	.45	.04	.41		Item28	.10	.52	.28	.01	.27
	Item30	.25	.50	.32	.06	.25		Item23	.25	.54	.35	.06	.29		Item29	.19	.53	.32	.04	.29
	**Father**							**Father**							**Father**					
	Item13	-	-	-	-	-		Item01	-	-	-	-	-		Item03	-	-	-	-	-
	Item15	.33	.43	.29	.11	.18		Item02	-	-	-	-	-		Item04	-	-	-	-	-
	Item17	.20	.51	.31	.04	.26		Item06	-	-	-	-	-		Item05	-	-	-	-	-
	Item19	.25	.66	.50	.06	.44		Item07	.10	.68	.47	.01	.46		Item10	.07	.64	.41	.00	.41
	Item20	.06	.63	.39	.00	.39		Item08	-	-	-	-	-		Item11	.10	.61	.38	.01	.37
	Item22	.16	.64	.43	.03	.41		Item09	.06	.71	.51	.00	.50		Item16	.38	.60	.50	.15	.36
	Item24	.17	.59	.38	.03	.35		Item12	.09	.63	.41	.01	.40		Item18	.20	.62	.42	.04	.38
	Item26	.09	.59	.35	.01	.34		Item14	.25	.71	.57	.06	.51		Item25	.18	.72	.55	.03	.52
	Item27	.22	.64	.46	.05	.41		Item21	.25	.58	.39	.06	.33		Item28	-.05	.54	.29	.00	.29
	Item30	.24	.57	.38	.06	.32		Item23	.18	.51	.29	.03	.26		Item29	.07	.63	.40	.00	.39

Mother as Reference Method

		T	M	1	2	3			T	M	1	2	3			T	M	1	2	3
Social Competence	**Teacher**						Anxiety-Withdraw	**Teacher**						Anger-Aggressive	**Teacher**					
	Item13	-	-	-	-	-		Item01	-	-	-	-	-		Item03	-	-	-	-	-
	Item15	.19	.63	.43	.04	.40		Item02	-	-	-	-	-		Item04	-	-	-	-	-
	Item17	.32	.68	.57	.10	.46		Item06	-	-	-	-	-		Item05	-	-	-	-	-
	Item19	.29	.68	.55	.08	.47		Item07	.10	.78	.61	.01	.60		Item10	.13	.78	.62	.02	.60
	Item20	.12	.70	.51	.02	.49		Item08	-	-	-	-	-		Item11	.18	.78	.64	.03	.61
	Item22	.32	.68	.56	.10	.46		Item09	.09	.88	.79	.01	.78		Item16	.42	.79	.80	.17	.63
	Item24	.25	.77	.65	.06	.59		Item12	.20	.84	.74	.04	.70		Item18	.33	.82	.79	.11	.68
	Item26	.14	.61	.39	.02	.37		Item14	.18	.85	.75	.03	.72		Item25	.34	.84	.82	.12	.70
	Item27	.23	.79	.68	.05	.63		Item21	.29	.63	.49	.08	.40		Item28	.27	.83	.77	.07	.70
	Item30	.18	.67	.49	.03	.45		Item23	.21	.65	.47	.04	.42		Item29	.38	.81	.80	.15	.66
	**Father**							**Father**							**Father**					
	Item13	-	-	-	-	-		Item01	-	-	-	-	-		Item03	-	-	-	-	-
	Item15	.54	.51	.55	.30	.26		Item02	-	-	-	-	-		Item04	-	-	-	-	-
	Item17	.52	.47	.48	.27	.22		Item06	-	-	-	-	-		Item05	-	-	-	-	-
	Item19	.67	.45	.64	.44	.20		Item07	.65	.43	.60	.42	.19		Item10	.52	.52	.54	.27	.27
	Item20	.66	.31	.53	.44	.09		Item08	-	-	-	-	-		Item11	.56	.43	.50	.32	.18
	Item22	.63	.44	.59	.40	.19		Item09	.58	.51	.60	.34	.26		Item16	.64	.42	.59	.41	.18
	Item24	.63	.36	.53	.40	.13		Item12	.53	.58	.62	.28	.33		Item18	.55	.53	.58	.30	.28
	Item26	.68	.33	.56	.46	.11		Item14	.53	.67	.73	.28	.44		Item25	.64	.53	.69	.41	.28
	Item27	.62	.51	.64	.39	.26		Item21	.54	.47	.51	.29	.22		Item28	.37	.52	.41	.13	.27
	Item30	.63	.43	.58	.40	.18		Item23	.56	.38	.46	.31	.14		Item29	.53	.53	.56	.28	.28

Father as Reference Method

		T	M	1	2	3			T	M	1	2	3			T	M	1	2	3
Social Competence	**Teacher**						Anxiety-Withdraw	**Teacher**						Anger-Aggressive	**Teacher**					
	Item13	-	-	-	-	-		Item01	-	-	-	-	-		Item03	-	-	-	-	-
	Item15	.15	.62	.41	.02	.39		Item02	-	-	-	-	-		Item04	-	-	-	-	-
	Item17	.28	.68	.54	.08	.47		Item06	-	-	-	-	-		Item05	-	-	-	-	-
	Item19	.25	.69	.54	.06	.48		Item07	.11	.78	.61	.01	.60		Item10	.01	.78	.61	.00	.61
	Item20	.12	.69	.49	.01	.48		Item08	-	-	-	-	-		Item11	.07	.78	.61	.00	.61
	Item22	.29	.68	.55	.08	.47		Item09	.05	.89	.80	.00	.80		Item16	.30	.83	.79	.09	.69
	Item24	.23	.77	.65	.05	.60		Item12	.22	.83	.74	.05	.69		Item18	.20	.85	.76	.04	.72
	Item26	.11	.61	.39	.01	.38		Item14	.19	.84	.75	.04	.71		Item25	.21	.86	.79	.05	.74
	Item27	.20	.80	.68	.04	.64		Item21	.30	.63	.48	.09	.39		Item28	.14	.84	.73	.02	.71
	Item30	.12	.70	.51	.01	.49		Item23	.25	.65	.48	.06	.42		Item29	.21	.84	.75	.04	.71
	**Mother**							**Mother**							**Mother**					
	Item13	-	-	-	-	-		Item01	-	-	-	-	-		Item03	-	-	-	-	-
	Item15	.65	.20	.46	.42	.04		Item02	-	-	-	-	-		Item04	-	-	-	-	-
	Item17	.64	.43	.59	.40	.19		Item06	-	-	-	-	-		Item05	-	-	-	-	-
	Item19	.72	.37	.66	.52	.14		Item07	.64	.39	.56	.41	.15		Item10	.53	.43	.46	.28	.18
	Item20	.68	.37	.59	.46	.14		Item08	-	-	-	-	-		Item11	.57	.47	.54	.32	.22
	Item22	.58	.47	.56	.34	.22		Item09	.57	.62	.71	.32	.39		Item16	.60	.66	.80	.36	.44
	Item24	.65	.56	.73	.42	.31		Item12	.57	.52	.60	.33	.27		Item18	.51	.55	.57	.26	.31
	Item26	.64	.37	.55	.41	.14		Item14	.55	.75	.86	.30	.56		Item25	.58	.45	.54	.33	.20
	Item27	.62	.41	.56	.39	.17		Item21	.57	.51	.57	.32	.26		Item28	.47	.40	.38	.22	.16
	Item30	.51	.57	.58	.26	.32		Item23	.63	.38	.54	.40	.14		Item29	.55	.37	.44	.30	.14

Notes: N=369. Using M8 with 22 itens. T= Trait Loading, M= Method Loading; 1=Reliability Coefficient, 2= Consistency Coefficient, 3= Method Specific Coefficient.

Suplementary table 2. Standardized Covariances of the Trait Factors in the CT-C(M-1) Model

Teachers as Reference Method

		SC									AW						AA					
		15	17	19	20	22	24	26	27	30	7	9	12	14	21	23	10	11	16	18	25	28
SC	17	.70																				
	19	.48	.73																			
	20	.72	.65	.56																		
	22	.49	.48	.56	.71																	
	24	.50	.62	.51	.53	.53																
	26	.29	.49	.44	.50	.36	.50															
	27	.52	.60	.56	.75	.63	.78	.65														
	30	.43	.73	.64	.59	.68	.81	.72	.86													
AW	7	-.15	-.38	-.18	.08	-.15	-.18	-.27	-.04	-.13												
	9	-.33	-.85	-.51	-.19	-.24	-.36	-.29	-.28	-.57	.99											
	12	-.16	-.51	-.20	.08	-.07	-.16	-.16	-.09	-.18	.84	1.16										
	14	-.03	-.64	-.37	.02	-.26	-.34	-.42	-.34	-.37	.75	1.14	.96									
	21	-.10	-.26	-.21	-.04	-.21	-.13	-.22	-.16	-.34	.42	.85	.65	.58								
	23	-.06	-.32	-.18	.15	-.03	-.21	-.23	-.13	-.30	.60	.85	.84	.67	.58							
AA	10	-.51	-.33	-.30	-.74	-.63	-.60	-.33	-.58	-.45	.10	.44	-.06	.22	.06	-.33						
	11	-.20	-.07	.05	-.28	-.32	-.32	-.08	-.25	-.17	.06	.08	-.03	.07	.04	-.22	.90					
	16	-.31	-.24	-.19	-.47	-.46	-.37	-.31	-.33	-.34	.06	.14	-.03	.14	-.07	-.10	.87	.56				
	18	-.33	-.32	-.21	-.49	-.48	-.36	-.27	-.32	-.28	.02	.12	-.08	.07	-.06	-.13	.98	.73	.79			
	25	-.36	-.40	-.32	-.48	-.47	-.54	-.42	-.54	-.52	.15	.26	.14	.34	.06	-.01	.77	.60	.68	.71		
	28	-.30	-.39	-.15	-.51	-.56	-.62	-.49	-.61	-.37	.30	.10	.20	.41	.00	.00	.99	.78	.75	.74	.90	
	29	-.34	-.31	-.15	-.41	-.39	-.44	-.34	-.46	-.25	.16	.07	.04	.25	.03	-.16	.71	.59	.58	.63	.63	.89

Mothers as Reference Method

		SC									AW						AA					
		15	17	19	20	22	24	26	27	30	7	9	12	14	21	23	10	11	16	18	25	28
SC	17	.65																				
	19	.41	.58																			
	20	.41	.37	.51																		
	22	.46	.41	.55	.44																	
	24	.39	.66	.42	.36	.56																
	26	.06	.16	.38	.63	.37	.31															
	27	.30	.46	.53	.49	.47	.49	.74														
	30	.17	.51	.42	.33	.56	.55	.51	.66													
AW	7	.06	.03	-.07	-.07	.05	-.09	.09	.17	.12												
	9	.05	-.03	-.09	-.05	-.05	-.14	.06	.05	-.05	.67											
	12	-.14	-.19	-.13	.01	-.16	-.09	.05	.00	-.01	.33	.47										
	14	-.05	-.20	-.07	.02	.02	-.14	.10	.07	-.07	.53	.70	.59									
	21	-.06	-.07	-.09	.06	-.12	-.18	.00	-.03	-.20	.49	.54	.54	.57								
	23	-.05	-.10	-.03	.00	-.06	-.21	-.02	.08	-.07	.42	.46	.29	.45	.47							
AA	10	-.07	.01	-.01	-.32	.00	-.11	.00	-.08	-.01	.27	.33	.22	.32	.23	.02						
	11	-.16	.03	.12	-.14	-.11	-.05	.11	.09	.05	.18	.25	.28	.31	.19	.13	.65					
	16	-.12	-.15	-.16	-.27	-.24	-.19	-.13	-.15	-.21	.10	.06	.16	.31	.31	.13	.43	.44				
	18	-.08	-.12	-.05	-.28	-.16	-.10	.04	.04	-.07	.10	.17	.09	.24	.18	.11	.52	.54	.72			
	25	-.20	-.23	-.20	-.47	-.25	-.31	-.25	-.22	-.32	.13	.16	.17	.34	.26	.16	.63	.40	.56	.51		
	28	.03	.08	.06	-.15	-.09	-.01	-.02	.13	.03	.23	.12	.28	.28	.38	.26	.43	.52	.43	.35	.41	
	29	-.11	-.07	-.05	-.26	-.10	-.10	-.09	-.09	-.02	.28	.22	-.01	.32	.11	.17	.51	.43	.41	.43	.41	.63

Fathers as Reference Method

		SC									AW						AA					
		15	17	19	20	22	24	26	27	30	7	9	12	14	21	23	10	11	16	18	25	28
SC	17	.82																				
	19	.50	.61																			
	20	.43	.37	.53																		
	22	.51	.41	.56	.44																	
	24	.44	.62	.40	.32	.53																
	26	.07	.14	.40	.61	.36	.27															
	27	.42	.49	.59	.50	.48	.48	.72														
	30	.24	.45	.40	.32	.50	.45	.44	.56													
AW	7	.07	.01	-.12	-.09	.05	-.16	.05	.12	.07												
	9	.07	-.06	-.16	-.11	-.07	-.23	-.05	-.01	-.04	.65											
	12	-.11	-.26	-.22	-.05	-.19	-.19	-.03	-.03	-.03	.32	.46										
	14	.01	-.29	-.16	-.02	-.03	-.28	-.06	.00	-.11	.56	.63	.62									
	21	-.08	-.14	-.16	-.03	-.21	-.26	-.09	-.10	-.23	.50	.47	.57	.52								
	23	-.06	-.17	-.08	-.04	-.11	-.30	-.07	.03	-.13	.43	.44	.31	.46	.51							
AA	10	-.03	.00	-.01	-.31	.02	-.13	-.01	-.07	-.05	.26	.24	.18	.28	.20	.02						
	11	-.17	-.05	.10	-.12	-.14	-.11	.06	.02	-.04	.16	.21	.26	.26	.15	.13	.61					
	16	-.09	-.12	-.13	-.18	-.17	-.18	-.13	-.14	-.21	.05	-.01	.09	.11	.21	.11	.39	.36				
	18	.00	-.12	.00	-.19	-.09	-.10	.06	.06	-.06	.04	.07	-.03	.10	.11	.08	.54	.52	.59			
	25	-.14	-.20	-.15	-.38	-.16	-.26	-.20	-.15	-.22	.14	.12	.16	.24	.18	.16	.63	.37	.46	.46		
	28	.10	-.01	.05	-.16	-.08	-.12	-.07	.07	-.03	.31	.12	.32	.33	.43	.35	.45	.46	.41	.44	.49	
	29	-.08	-.11	-.06	-.25	-.10	-.14	-.10	-.11	-.11	.30	.18	.01	.30	.09	.16	.56	.41	.32	.41	.48	.72

Note: SC= Social Competence, AW= Anxiety-Withdrawal, AA=Anger-Angressive; M= Mother, F= Father, T=Teacher
